# Evaluation of Glycyrrhizic Acid Therapeutic Effect and Safety in *Mycoplasma gallisepticum* (HS Strain)-Infected Arbor Acres Broilers

**DOI:** 10.3390/ani12101285

**Published:** 2022-05-17

**Authors:** Fuli Hu, Ronglong Luo, Shuwen Duan, Qiao Guo, Lulu Wang, Guangyang Jiang, Changyong Fan, Mengyun Zou, Tengfei Wang, Yingjie Wang, Yingfei Sun, Xiuli Peng

**Affiliations:** Key Laboratory of Agricultural Animal Genetics, Breeding and Reproduction, Ministry of Education, College of Animal Science and Technology and College of Veterinary Medicine, Huazhong Agricultural University, Wuhan 430070, China; hufuli2005@163.com (F.H.); luoronglong@webmail.hzau.edu.cn (R.L.); duanshuwen@webmail.hzau.edu.cn (S.D.); guo_qiao@webmail.hzau.edu.cn (Q.G.); wanglulu@webmail.hzau.edu.cn (L.W.); jh11111204@163.com (G.J.); fanjuangjun888@163.com (C.F.); zoumengyun@webmail.hzau.edu.cn (M.Z.); wangtengfei@webmail.hzau.edu.cn (T.W.); wang985@webmail.hzau.edu.cn (Y.W.); sunyingfei@webmail.hzau.edu.cn (Y.S.)

**Keywords:** Arbor Acres (AA) broiler, Glycyrrhizic acid, *Mycoplasma gallisepticum*, production performance, serum biochemical index

## Abstract

**Simple Summary:**

*Mycoplasma gallisepticum* (MG) can induce chronic respiratory disease (CRD) in chickens. Glycyrrhizic acid (GA) is the major ingredient of licorice and shows excellent anti-pathogenic microorganism and anti-inflammatory properties. Based on above, this study has evaluated the therapeutic effects and safety of GA in MG-infected broilers. Study results showed that GA could inhibit the proliferation of MG in vitro and expression of MG adhesion protein (pMGA1.2) in broiler lungs. GA restored production performances and attenuated MG-induced organ damage and abnormal biochemical indicator changes in MG-infected broilers. In conclusion, GA displayed significant therapeutic efficacy regarding MG infection and had no adverse effects on broilers (100 mg/kg/d).

**Abstract:**

This study was conducted to evaluate the therapeutic effects and safety of GA in MG-infected broilers. Our results showed that the minimum inhibitory concentration of GA was 31.25 μg/mL. Moreover, GA inhibited the expression of MG adhesion protein (pMGA1.2) in the broilers’ lungs. GA treatment clearly decreased the morbidity of CRD and mortality in the MG-infected broilers. Compared with the model group, GA treatment significantly decreased gross air sac lesion scores and increased average weight gain and feed conversion rate in the MG-infected broilers. Histopathological examination showed GA treatment attenuated MG-induced trachea, immune organ and liver damage in the broilers. Moreover, GA treatment alone did not induce abnormal morphological changes in these organs in the healthy broilers. Compared with the model group, serum biochemical results showed GA treatment significantly decreased the content of total protein, albumin, globulin, alanine aminotransferase, aspartate aminotransferase, total bilirubin, creatinine, uric acid, total cholesterol, and increased the content of albumin/globulin, alkaline phosphatase, apolipoprotein B and apolipoprotein A-I. In conclusion, GA displayed a significant therapeutic efficacy regarding MG infection and had no adverse effects on the broilers (100 mg/kg/d).

## 1. Introduction

*Mycoplasma gallisepticum* (MG) is one of the most pathogenic mycoplasmas of poultry, and has a world-wide distribution [1]. MG is the primary aetiologic agent of infectious sinusitis in game birds, turkeys, passerine birds and pigeons and chronic respiratory disease (CRD) in chickens of all ages [2]. MG infection in chicken farms usually lasts for a long time, is very difficult to be eliminated completely, and leads to serious economic losses in the poultry industry in terms of low carcass quality, reduced feed conversion efficiency, egg production and hatchability [3,4,5]. MG adheres the surface of ciliated epithelial cells of the respiratory tract, followed by a subsequent colonization in host cells and production of vigorous inflammatory responses [6]. A previous study has demonstrated that the adhesin protein pMGA1.2 of MG is required for MG to adhere in chickens, and pMGA1.2 specifically binds to host cell apolipoprotein A-I (ApoA-I) to elicit inflammation [7]. Moreover, chicken ApoA-I has been expressed in many tissues, including the liver, lung, kidneys, heart, glandular stomach, duodenum, bursa of Fabricius, and thymus [8].

Antibiotic treatment (Tiamulin) is currently one of most important choices for the control of MG infection in the poultry industry [9,10]. However, antibiotic usage in animals may result antibiotic residues in foodstuffs such as eggs and meat [11]. Furthermore, these antibiotic residues may cause various side effects such as the transfer of antibiotic-resistant bacteria to humans, immunopathological effects, allergies, mutagenicity, nephropathy, hepatotoxicity, reproductive disorders, bone marrow toxicity and even carcinogenicity [12,13]. Above all, the increase in antibiotic resistance is a global concern for human and animal health and resistant microorganisms can spread between food-producing animals and humans [14]. Therefore, a safer and more effective alternative therapy for MG infection is urgently required.

Licorice, the root of *Glycyrrhiza* *glabra*, is used as a traditional herbal medicine in Asian countries [15]. The pharmacological effects of licorice are attributed to its various metabolites including glycyrrhizic acid (GA) and glycyrrihetic acid (glycyrrhetinic acid) [16]. GA is absorbed through the intestine, transported to the liver, metabolized into glucuronide and sulfate conjugates through phase II biotransformation, and excreted to the biliary system [17]. Numerous studies have provided scientific evidence related to the pharmacological properties of GA including anti-asthmatic, hepatoprotective, antioxidative, anti-inflammatory, antimicrobial and immunoregulatory effects [18,19,20]. Dipotassium glycyrrhizinate (DG) exhibits significant inhibitory activity against Marek’s disease virus (MDV) in chicken embryo fibroblast cells [21]. Moreover, GA could enhance *Salmonella*-killing capacity in the chicken macrophages [22]. Furthermore, GA could alleviate LPS-induced acute lung injury in the mice [23]. However, the possible therapeutic effects on MG infection and side effects of GA have been not reported in broilers. The purpose of this study was to evaluate the possible therapeutic effects and safety of GA in MG-infected broilers.

## 2. Materials and Methods

### 2.1. Ethical Considerations

This study was performed at Huazhong Agricultural University. The experimental protocol was in accordance with the animal care and use committee guidelines of our University (Ethics Approval Code: HZAUCH-2020-0011), and followed national laws and National Research Council (NRC) guidelines for the care and use of laboratory animals.

### 2.2. Chemicals and Reagents

**Figure 1:**
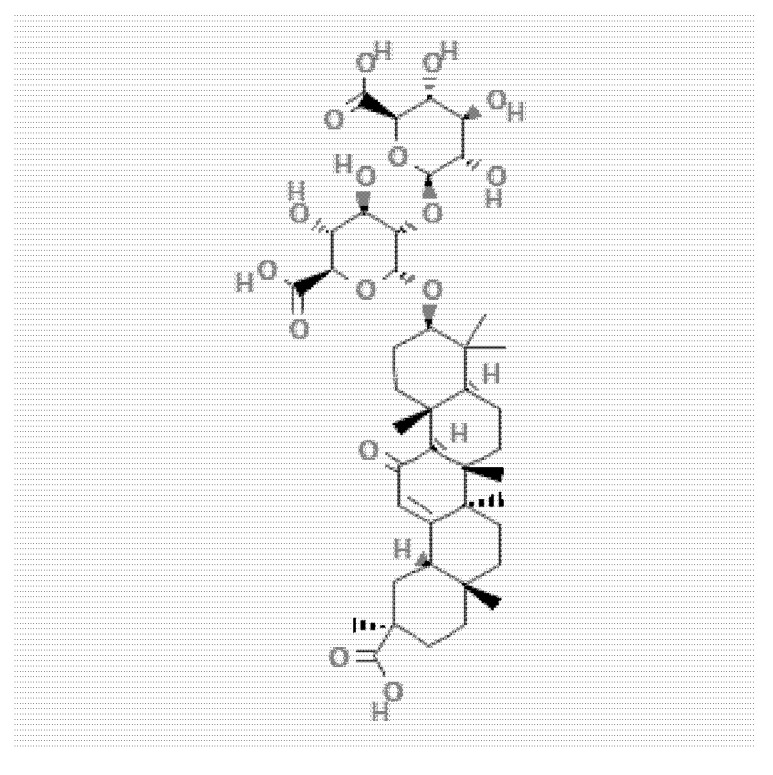
The structural of GA (PubChem CID 14982, accessed on 14 May 2022).

Glycyrrhizic acid (GA) (purity ≥ 98%) was purchased from the Lansley Technology Co., Ltd. (Luoyang, China). Figure 1 shown the chemical structure of GA. Total protein (TP), albumin (ALB), alanine aminotransferase (ALT), aspartate aminotransferase (AST), alkaline phosphatase (ALP), total bilirubin (TBIL), urea nitrogen (Urea), creatinine (Cr), uric acid (UA), glucose (GLU), total cholesterol (TCHO), apolipoprotein B (ApoB), triglyceride (TG), apolipoprotein A-I (ApoA-I) test kits were purchased from the Senlong Biological Technology Co., Ltd. (Zhuhai, China).

### 2.3. Mycoplasma Gallisepticum HS Strain (MG) Culture

*Mycoplasma Gallisepticum* HS strain (MG) is a virulent strain that was isolated from a chicken farm (Hubei, China) and provided by the State Key Laboratory of Agricultural Microbiology of Huazhong Agricultural University (Wuhan, China). MG was multiplied in improved FM-4 medium with 12% inactivated swine serum. A color-changing unit (CCU) assay was applied to examine the vitality of MG in liquid medium [24].

### 2.4. Minimum Inhibitory Concentration (MIC)

A suspension 1 × 10^4^ CCU/mL of *Mycoplasma Gallisepticum* HS strain (MG) was used in order to evaluate the MIC of GA. The MIC of GA was determined by 2-fold serial dilution method. Briefly, GA was dissolved using FM-4 medium containing 0.2% dimethyl sulfoxide (DMSO), then we serially diluted it by 1:2 each time. Next, 0.1 mL of MG (1 × 10^4^ CCU/mL) was added to these tubes. These tubes were incubated in a constant temperature incubator at 37 °C until MG-positive control group culture medium turned yellow. The minimum GA concentration that did not change color was the MIC.

### 2.5. Experimental Design

A total of 150 one-day-old Arbor Acres (AA) broilers were reared for 12 days prior to experiments to allow them to adapt to experimental conditions. Broilers were reared in a closed room to regulate light and temperature during the experiment. The animal density was the same for each experimental group in the experiment. Ad libitum feed and water were provided to broilers. After 12 days, broilers were wing-tagged and randomly divided into 5 experimental groups with 3 replicates per group and 10 broilers per replicate. The ratios of male and female broilers in each replicate were same. Experimental groups including A: control group, B: GA-alone group (100 mg/kg), C: model group (control MG infection group), D: tiamulin (Tia) therapy group (500 mg/L), E: GA therapy group (100 mg/kg). The C, D and E groups were challenged with 0.5, 0.2 and 0.3 mL of MG at a concentration of 1 × 10^9^ CCU/mL into the right air sacs in the thoracic region, eye and nasal cavity for 5 days, respectively [25]. After 5 days of challenge, all broilers had obvious symptoms of CRD. GA was given once in a day in the B and E groups and the D group was treated with Tia. After 7 days of GA or Tia treatment, broilers were humanely euthanized to avoid suffering, then blood samples, tracheal tissue, lung, thymus, spleen, bursa of Fabricius and liver were collected for further experimental analyses.

### 2.6. Morbidity, Cure Rate and Mortality

After broilers were challenged with MG for 5 days, we recorded the morbidity of CRD in every experimental group. Nine experts who did not know the experimental grouping made their judgments based on whether broiler had typical CRD symptoms. If more than two-thirds of the experts thought that broilers had symptoms of CRD, such as sneezing, runny nose, cough, redness and swelling around the eyes, lethargy and listlessness, we concluded that the broiler was positive individual of CRD. The morbidity of CRD was calculated based on broilers with obvious symptoms/all broilers in every experimental group. If more than two-thirds of experts thought that the broilers lost the symptoms of CRD after treatment with GA or Tia, we concluded that the broiler had been successfully cured. Then, the cure rate was calculated based on cured broilers/infected broilers. The mortality of broilers was calculated in every experimental group. It is worth mentioning that if broilers died within the first day of treatment, we did not count these broilers as deaths during treatment period but as deaths during the infection period; these were not used in the calculation of cure rate.

### 2.7. Body Weight and Feed Efficiency

The live weight of each broiler was measured with an electronic scale at 13 d of age, 18 d of age and 24 d of age. We recorded the feed intake at 13–17 days and 18–24 days. Then, the average feed intake (AFI_13-17_ and AFI_18-25_) and average body weight gain (AWG_13-17_ and AWG_18-25_) were calculated. The feed conversion ratio (FCR) is the net feed consumption of broiler unit weight gain. The FCR for each individual was estimated based on the ratio between unit weight gain and feed consumption. The FCR was calculated as follows: FCR = AFI/AWG. Where, FCR_13-17_ = AFI_13-17_/AWG_13-17_, FCR_18-25_ = AFI_18-25_ /AWG_18-25_.

### 2.8. Gross Air Sac Lesion Scores (ALS) and Injury Reduction Rate (IRR)

After broilers were euthanized, we dissected each broiler and observed the air sacs and scored air sac lesions. All air sac lesion results were scored in a blinded manner. The gross air sac lesion was scored on a scale from 0 to 4, referencing the modification standard described previously [26,27]: 0 = no significant changes; 1 = only slightly thickened air sac or yellow oozing spots; 2 = some yellow oozing spots and thickened air sac; 3 = a large number of yellow-white lesions with caseous exudates and thickened air sac; 4 = almost covered with yellow-white lesions with caseous exudates and thickened air sac. Injury reduction rate = (average air sac lesion score of control MG-infection group-average air sac lesion score of treatment group)/average air sac lesion score of control MG-infection group × 100%.

### 2.9. Immune Organ Index

After broilers were euthanized, thymus, spleen and bursa of Fabricius were collected. All immune organ weight measurements were acquired using an electronic balance with a precision of 0.01 g. The immune organ indices (g/kg) were calculated by the weight (g) of thymus, spleen and bursa of Fabricius/body weight (kg).

### 2.10. Histopathological Examination

Samples from tracheal tissue, liver and immune organs were collected and fixed in 10% formalin for at least 24 h and dehydrated and embedded in wax. Sections (5 μm) were stained with Hematoxylin and Eosin strain (H & E strain) for histopathological examination. The tracheal mucosal thickness was measured at four each sections to obtain the mean tracheal mucosal thickness for each broiler [28]. Four splenic corpuscles were randomly selected for each specimen, then the radius of the splenic corpuscle (R) was calculated for each splenic corpuscle. The area of the splenic corpuscle was calculated using πR^2^. Finally, the mean area of the splenic corpuscle for each specimen was calculated.

### 2.11. Serological Analysis

Blood samples were collected and serum biochemical parameters were measured using the Dongtang Biochemical Analyzer 8018 (Guangzhou Dongtang Electronic Technology Co., Ltd., Guangzhou, China). These serum biochemical parameters included TP (g/L), ALB (g/L), ALT (U/L), AST (U/L), ALP (U/L), TBIL (umol/L), Urea (mmol/L), Cr (umol/L), UA (umol/L), GLU (mmol/L), TCHO (mmol/L), ApoB (mg/L), TG (mmol/L), ApoA-I (mg/L). Globulin (GLB) (g/L) = TP-ALP. The A/G ratio = ALB/GLB.

### 2.12. Quantitative Real-Time Polymerase Chain Reaction (qRT-PCR)

Total RNA was extracted from broilers’ lungs using Trizol Reagent (TIANGEN, Beijing, China). The quality of RNA was evaluated by measuring the absorbance at a 260/280 ratio. cDNA was synthesized using the cDNA Synthesis SuperMix (Vazyme, Nanjing, China) according to the manufacturer instructions. The mRNA expression levels of pMGA1.2 and GAPDH were determined using qRT-PCR (Bio-Rad, Hercules, CA, USA) with SuperReal PreMix Plus SYBR Green (Yeasen, Shanghai, China). The total volume of the reaction was 10 μL and the thermocycler program was as follows: 95 °C for 5 min, 40 cycles of 95 °C for 10 s, 60 °C for 20 s, and 72 °C for 20 s. The relative mRNA levels were calculated using the 2^−^^ΔΔCt^ method. GAPDH was used as an internal control. The primers used in this study are listed in Table 1.

### 2.13. Statistical Analysis

The experimental study considered a completely randomized design. To determine the data normality and the variance uniformity, the Kolmogorov–Smirnov and Bartlett tests were used. Next, the data were processed by a simple classification analysis of variance (ANOVA). Where necessary, a post hoc analysis (Duncan) was used. All data were analyzed using SPSS 23.0 Analyst (Chicago, IL, USA). All graphs were made using GraphPad prism 8.0 (San Diego, CA, USA). Differences were considered significant at *p* value ≤ 0.05.

## 3. Results

### 3.1. GA Significantly Inhibited pMGA1.2 Expression

**Figure 2:**
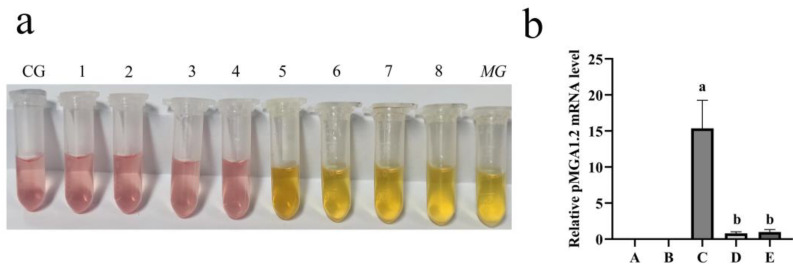
GA inhibited MG proliferation in vitro and pMGA1.2 expression in the broilers’ lungs. (**a**) GA inhibited the proliferation of MG in vitro. CG: control group; 1: 250.00 μg/mL; 2: 125.00 μg/mL; 3:62.50 μg/mL; 4: 31.25 μg/mL; 5: 15.63 μg/mL; 6: 7.81 μg/mL; 7: 3.91 μg/mL; 8: 1.96 μg/mL; MG: MG-positive control group. (**b**) GA inhibited pMGA1.2 expression in the broilers’ lungs. A: control group, B: GA-alone treatment group, C: model group, D: Tia therapy group, E: GA therapy group. Bars with different lowercase letters represent significant differences (*p* ≤ 0.05). Values represent the mean ± SD (*n* = 3).

As shown in Figure 2a, glycyrrhizic acid (GA) dose-dependently inhibited MG proliferation in vitro and the MIC of GA was 31.25 μg/mL. The qRT-PCR result showed that GA treatment significantly inhibited pMGA1.2 expression in the broilers’ lungs compared with the model group (*p* < 0.001) (Figure 2b)**.**

### 3.2. GA Restored the Production Performances of MG-Infected Broilers

The morbidity of CRD was 90.00% in the model group. The cure rates in the Tia therapy group and GA therapy group were 91.67% and 92.59%, respectively. The mortality rates of broilers in the model group, Tia therapy group and GA therapy group were 16.67%, 10.00% and 3.33%, respectively (Table 2). MG infection significantly decreased AWG (*p* < 0.001) and feed efficiency (*p* = 0.008) of the MG-infected groups compared with the control group at 18–24 d, but GA treatment significantly restored AWG (*p* = 0.007) and feed efficiency (*p* = 0.023) compared with the model group (Table 3).

### 3.3. GA Attenuated MG-Infected Broilers’ Air Sac Lesions

Air sac lesion results showed that GA treatment significantly attenuated MG-induced gross air sac lesion score compared with the model group in the broilers (*p* < 0.001) (Table 4).

### 3.4. GA Decreased MG-Induced Mean Tracheal Mucosal Thickness

Histopathological examination showed that GA treatment did not increase mean tracheal mucosal thickness compared with the control group (*p* = 0.935). MG infection significantly increased mean tracheal mucosal thickness compared with the control group (*p* < 0.001), but GA interference significantly decreased mean tracheal mucosal thickness compared with the model group in the broilers (*p* < 0.001) (Table 5).

### 3.5. GA Attenuated MG-Induced Immune Organ Damage

**Figure 3:**
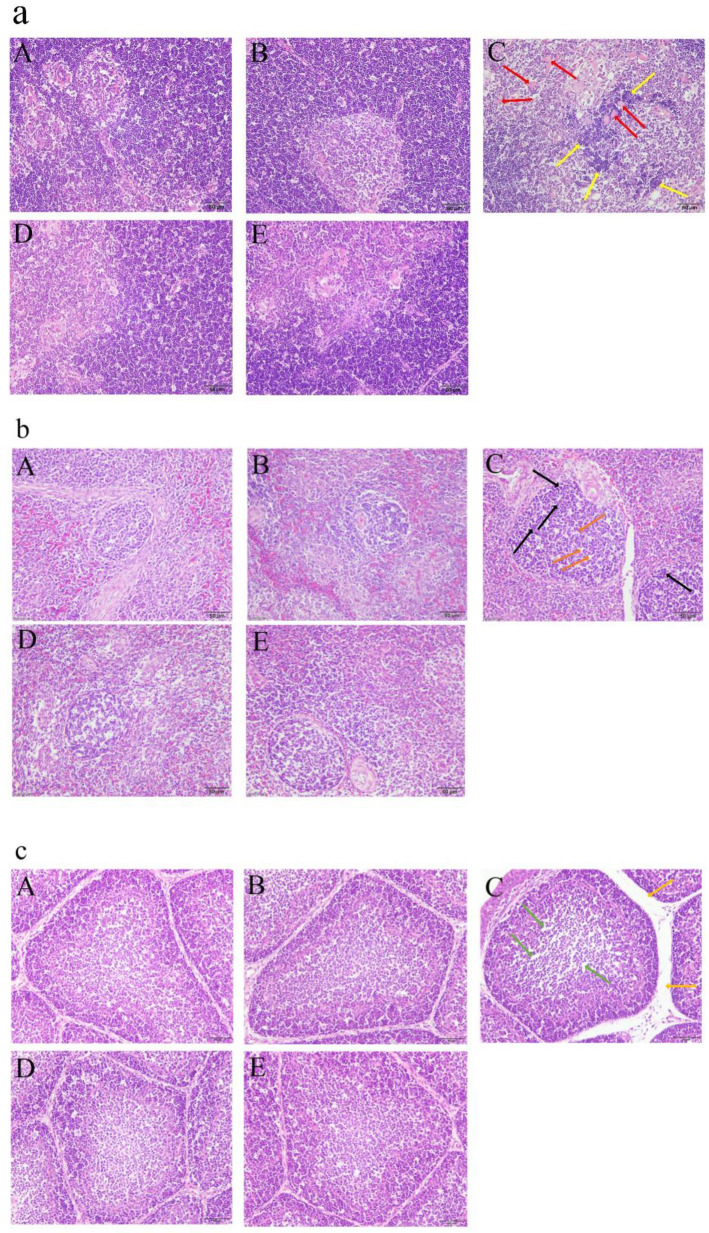
GA alleviated MG-induced immune organ damage in the broilers. (**a**) GA alleviated MG-induced thymus damage. Yellow arrows show inflammatory cells’ infiltration and red arrows show vacuoles in the cortex and medulla of thymus. (**b**) GA alleviated MG-induced spleen damage. Orange arrows shows lymphocytes’ shedding and black arrows show mononuclear hyperplasia. (**c**) GA alleviated MG-induced bursa of Fabricius damage. Green arrows show lymphocytes’ shedding and golden arrows show increased space between bursal follicles. (**A**): control group, (**B**): GA-alone treatment group, (**C**): model group, (**D**): Tia therapy group, (**E**): GA therapy group.

MG infection significantly increased thymus (*p* < 0.001), spleen (*p* = 0.002) and bursa indexes (*p* < 0.001), but GA treatment significantly decreased MG-induced thymus (*p* < 0.001), spleen (*p* = 0.005) and bursa indexes (*p* < 0.001) (Table 6). MG infection significantly increased the inflammatory cells’ infiltration and number of vacuoles in the thymus (Figure 3a(C)), the lymphocyte shedding and mononuclear hyperplasia in the spleen (Figure 3b(C)), and the lymphocytes shedding and space between bursal follicles in the bursa of Fabricius (Figure 3c(C)). However, GA treatment significantly attenuated these abnormal morphological changes in the thymus (Figure 3a(E)), spleen (Figure 3b(E)) and bursa of Fabricius (Figure 3c(E)). Moreover, GA treatment significantly decreased the area of splenic corpuscle compared with the model group (*p* = 0.005) (Table 7). GA-alone treatment did not induce abnormal histological changes in the thymus (Figure 3a(B)), spleen (Figure 3b(B)) or bursa of Fabricius (Figure 3c(B)) in the healthy broilers.

### 3.6. GA Attenuated MG-Induced Serum Biochemical Indexes Disorder

**Figure 4:**
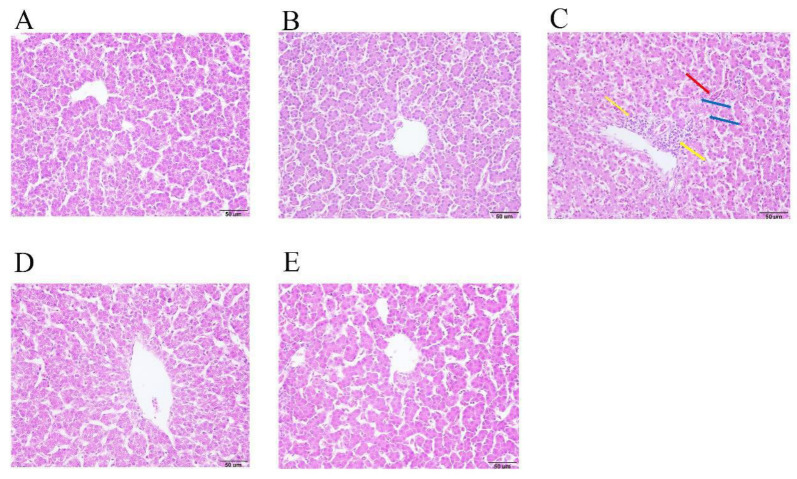
GA alleviated MG infection-induced liver damage in broilers. (**A**) control group, (**B**) GA-alone treatment group, (**C**) model group, (**D**) Tia therapy group, (**E**) GA therapy group. Yellow arrows show inflammatory cell infiltration around the central veins, blue arrows show vacuolar degeneration and red arrows show sinus congestion.

As presented in Table 8, serum biochemistry results show that MG infection significantly increased the content of TP (*p* < 0.001), ALB (*p* < 0.001), GLB (*p* < 0.001), ALT (*p* < 0.001), AST (*p* < 0.001), TBIL (*p* < 0.001), Cr (*p* < 0.001), UA (*p* = 0.001) and TCHO (*p* < 0.001) and decreased the content of ALP (*p* < 0.001), ApoB (*p* = 0.007), TG (*p* = 0.006), ApoA-I (*p* < 0.001) and A/G ratio (*p* < 0.001) in the serum compared with the control group. However, GA treatment significantly down-regulated the content of TP (*p* < 0.001), ALB (*p* < 0.001), GLB (*p* = 0.001), ALT (*p* = 0.004), AST (*p* < 0.001), TBIL (*p* = 0.010), Cr (*p* < 0.001), UA (*p* = 0.004), and TCHO (*p* < 0.001), and up-regulated the content of A/G ratio (*p* = 0.002), ALP (*p* < 0.001), ApoB (*p* = 0.011) and ApoA-I (*p* = 0.001) in the serum compared with the model group in the broilers. At the same time, GA-alone treatment significantly decreased the content of TG (*p* = 0.045) in the serum compared with the control group in healthy broilers. In addition, MG infection induced extensive inflammatory cell infiltration around the central veins and increased vacuolar degeneration, and the sinusoids were congested in the liver tissue. However, these pathological changes were significantly attenuated after GA treatment in the broilers (Figure 4).

## 4. Discussion

Glycyrrhizic acid (GA) is considered the most active moieties in licorice and exhibits many pharmacological effects, especially in inhibiting the reproduction of pathogenic bacteria. Dipotassium glycyrrhizinate (DG) exhibited significant inhibitory activity against Marek’s disease virus and infectious bursal disease virus (IBDV) in a dose-dependent manner. The 50% effective concentration of DG was 893.5 mg/mL and 663.2 mg/mL, and selective index was ≥43.4 and ≥44.5, respectively [21,29]. GA could enhance *Salmonella*-killing capacity by enhancing the production of reactive oxygen and nitrogen species and increasing the expression of antimicrobial genes in chicken macrophages [22]. Our results showed that GA dose-dependently inhibited MG proliferation in vitro, and the MIC of GA was 31.25 μg/mL. Furthermore, GA significantly suppressed the expression of pMGA1.2 in the broilers’ lungs. A previous study has demonstrated that pMGA1.2 is a major adhesion protein of MG and is necessary for MG adhesion in chickens [7]. So, these results revealed that GA could inhibit the proliferation and adhesion of MG.

MG can induce CRD in chickens and infectious sinusitis in turkeys, and is regarded as one of the most cost infectious diseases in the poultry industry [9]. In this study, the morbidity of CRD and mortality in MG-infected broilers were 90.00% and 16.67%, respectively. However, 92.59% of MG-infected broilers lost the typical symptoms of CRD after being treated by GA. Some studies have revealed that MG infection can lead to a decrease in mean weight gain and poor feed efficiency in chickens [6,9]. Our data showed that MG infection significantly reduced AWG and feed efficiency of broilers at 18–24 days. However, when GA was applied, AWG and feed efficiency of MG-infected broilers were significantly restored compared with the model group at 18–24 days. Therefore, our results illuminated that GA could restore the production performances of MG-infected broilers.

MG infection can induce profound inflammatory responses in the chicken respiratory tract, including the trachea, air sacs and lungs [30]. Researchers have reported that MG infection can cause cloudy thickening of the air sac and yellowish foci [31]. GA alleviates acute lung injury induced by LPS by PI3K/AKT-suppressing macrophagic Nlrp3 inflammasome activation in mice [23]. Furthermore, GA has a protective effect against sepsis-induced acute lung injury by inhibiting the inflammatory response, damage from oxidative stress, and apoptosis via inactivation of NF-*κ*B and MAPK signaling pathways in rats [32]. In this study, GA treatment significantly attenuated MG-induced trachea and air sac damage. GA-alone treatment did not induce damage of these organs in healthy broilers. Moreover, previous research has revealed that GA can attenuate MG-induced inflammation and apoptosis through suppressing the MAPK pathway in chicken lungs [33]. So, GA may provide a protective role for the air sacs, trachea and lung tissue during MG infection in broilers.

The thymus, spleen and bursa of Fabricius are important immune organs of chickens and are closely linked to chickens’ immune function [34]. MG infection would induce immune organ injury, such as increasing cell necrosis and inflammation cell infiltrates in the thymus, increasing cell necrosis and lymphopenia in the spleen and increasing vacuolation and the space between bursal follicles in the bursa of Fabricius in chickens [35,36,37]. Hypertrophied spleens were observed in the LPS-challenged chicks, which may be because LPS-induced immune response increased proinflammatory cytokine production and simultaneously recruited inflammatory cells to the spleen, leading to compensatory splenic hyperplasia [38]. Tetramethyl thiuram disulfide (Thiram) significantly increases spleen index in chickens, which may be due to the compensatory increases seen in organs after body injury [39]. In this study, MG infection significantly increased the thymus, spleen and bursa indexes of broilers. Histopathological examination results revealed that MG induced thymus, spleen and bursa damage in the broilers. However, GA treatment significantly decreased the MG-induced thymus, spleen and bursa of Fabricius indexes and attenuated MG-induced immune organ damage. Furthermore, GA-alone treatment did not induce abnormal histopathologic changes in immune organs of healthy broilers. We speculate that the reason for the increased spleen index due to MG infection may be compensatory splenic hyperplasia in the broilers. However, more studies are needed to investigate the mechanism of MG in immune organ damage in broilers in the future. In conclusion, GA could attenuate MG-induced immune organs damage during MG infection in broilers.

One study found that avian hepatitis E 526 virus and avian leukosis virus subgroup J could induce hepatic injury in chickens [40]. Liver damage could be confirmed by detecting some relative hepatobiliary function indexes in the serum [41]. An increase in serum GLB and ALB concentrations and low A/G ratio are significantly associated with chronic damaged liver in rats [42]. The content of TBIL in the serum in autoimmune hepatitis patients is significantly higher compared with healthy people [43]. The activity of ALT and AST are sensitive indicators of acute hepatic necrosis and elevated activities of AST and ALT in the serum are indicative of cellular leakage and loss of functional integrity of the cell membrane in chickens [44]. ALP, an enzyme long associated with the mineralization process, is markedly increased at early sites of mineral formation in calcifying tissues in chickens [45]. ALP activity was influenced by Ca source and intestinal ALP activity enhancement could improve broiler bone characteristics [46]. ALP deficiency can occur in some bone-development-relevant diseases, such as rickets, osteomalacia and tooth sludge [47]. So, the decreased content of ALP in the serum would not be conducive to bone development. MG infection significantly increased the content of TP, ALB, GLB, ALT, AST, TBIL, Urea, Cr, UA and TCHO and decreased the content of ALP, ApoB, TG, ApoA1 and A/G ratio in the serum compared with the control group. However, GA treatment significantly decreased the content of TP, GLB, ALT, AST, TBIL, Cr, UA, and TCHO, and increased the A/G ratio, ALP, ApoB, TG and ApoA1 in the serum compared with the model group. At the same time, GA-alone treatment significantly decreased the content of TG in the serum compared with control group in the healthy broilers. Moreover, histopathological examination of liver showed that GA significantly alleviated MG-induced liver injury. A previous study found that compound ammonium glycyrrhizin protected chicken primary hepatocytes against damage induced by Ochratoxin A via anti-oxidative and anti-apoptosis mechanisms, which may be a potential candidate for the prevention and treatment of chicken liver injury [48]. From these results, we speculated that MG infection could induce abnormal bone development, but GA may have a potential benefit in promoting normal bone development in broilers. More experiments are needed to validate our hypothesis. In conclusion, GA could significantly attenuate MG-induced liver damage in broilers.

Infectious bronchitis virus (IBV), fowl adenovirus serotype 4 (FAdV-4) and avian orthoreovirus (ARV) would result in significantly severe lesions of the kidney of chickens [49,50]. The increases in UA, Cr and Urea content in the serum are significantly associated with renal injury in broilers [51]. Our results show that AG treatment significantly decreased the content of UA, Urea and Cr in the serum of MG-infected broilers. GA could attenuate sepsis-induced acute kidney injury by inhibiting the NF-*κ*B signaling pathway in rats [52]. Moreover, GA could improve renal injury and inflammatory responses in diabetic rats by regulating RAGE/TLR4-related ERK and p38 MAPK/NF-κB activation [53]. These results indicated that GA could effectively attenuate MG-induced renal damage in broilers.

Glucose and lipid metabolism disorder is associated with many diseases, such as hepatic function disorder and cardiovascular diseases [54,55]. TCHO concentration was significantly increased in a cardiac damage model induced by arsenic or/and copper in *Gallus gallus* [56]. ApoB is associated with the development of dyslipidemia and atherosclerosis, which could be used in diagnosis and therapy of the atherogenic dyslipoproteinemias [57,58]. ApoA-I has been demonstrated to be the receptor of MG adhesion protein pMGA1.2 in chickens [7]. In our study, MG infection significantly increased the concentration of CHTO and decreased the concentration of TG, ApoB and ApoA1 in the serum of MG-infected broilers. However, GA treatment significantly decreased the concentration of CHTO and increased the concentration of TG, ApoB and ApoA1 in the MG-infected broilers. Above all, GA significantly decreased the concentration of TG in the serum of healthy broilers. So, GA may protect the heart from damage and the atherogenic dyslipoproteinemias induced by MG infection in broilers.

## 5. Conclusions

GA could effectively inhibit the proliferation and adhesion of MG. Moreover, GA decreased the morbidity of CRD and mortality in MG-infected broilers. GA significantly recovered production performance and ameliorated MG-induced air sac, immune organ, trachea, liver and heart damage in the broilers. The appropriate dose of GA treatment, such as 100 mg/kg per day, had no obvious significant side effects in the broilers. So, GA may be used as an alternative to Tia in the prevention and treatment of MG infection in broilers.

## Figures and Tables

**Table 1:** Primers for quantitative qRT-PCR.

Name	Primer Sequence (5′-3′)	Accession No.
pMGA1.2-F	ACACCAACTCCAAACCCTG	AF275312_2
pMGA1.2-R	GATTGTCGCCCATCATAC	AF275312_2
GAPDH-F	GAGGGTAGTGAAGGCTGCTG	NM-204305
GAPDH-R	CACAACACGGTTGCTGTATC	NM-204305

**Table 2:** Morbidity of CRD, cure rate and mortality of broilers between different treatment groups.

Treatments					
Items	A	B	C	D	E
Morbidity (%)			90.00 (27/30)	90.00 (27/30)	93.33 (28/30)
Cure rate (%)				91.67 (22/24)	92.59 (25/27)
Mortality (%)	0	0	16.67 (5/30)	10.00 (3/30)	3.33 (1/30)

**Table 3:** Changes in production performance of broilers between different groups during the experiment.

Treatments							
Items	A	B	C	D	E	SEM±	*p*-Value
13–17 days							
AWG (g)	245.82 ^b^	241.84 ^b^	229.05 ^a^	232.91 ^a^	231.40 ^a^	1.266	<0.001
FCR	1.50	1.59	1.60	1.65	1.66	0.084	0.117
18–24 days							
AWG (g)	434.12 ^c^	411.96 ^c^	112.60 ^a^	338.82 ^b^	320.92 ^b^	12.603	<0.001
FCR	1.88 ^a^	1.89 ^a^	2.50 ^b^	2.01 ^a^	1.99 ^a^	0.079	0.044

^a,b,c^ Values with different lowercase letters represent significant differences (*p* ≤ 0.05).

**Table 4:** GA decreased MG-induced gross air sac lesion scores in the broilers.

Treatments							
Items	A	B	C	D	E	SEM ±	*p*-Value
ALS	0	0	3.30 ^c^	1.60 ^b^	1.05 ^a^	0.142	<0.001
IRR%	-	-	-	51.51%	68.18%		

^a,b,c^ Values with different lowercase letters represent significant differences (*p* ≤ 0.05) (*n* = 20).

**Table 5:** GA decreased MG-induced mean tracheal mucosal thickness in the broilers.

Treatments							
Items	A	B	C	D	E	SEM ±	*p*-Value
Thickness (μm)	43.82 ^a^	44.24 ^a^	195.32 ^d^	70.58 ^b^	130.42 ^c^	7.768	<0.001

^a,b,c,d^ Values with different lowercase letters represent significant differences (*p* ≤ 0.05) (*n* = 3).

**Table 6:** GA decreased MG-induced immune organ indexes in the broilers.

Treatments							
Items	A	B	C	D	E	SEM ±	*p*-Value
Thymus index	3.24 ^a^	3.04 ^a^	5.18 ^b^	3.08 ^a^	3.25 ^a^	0.126	<0.001
Spleen index	1.01 ^a^	1.03 ^a^	1.37 ^b^	0.90 ^a^	0.94 ^a^	0.031	<0.001
Bursa index	1.57 ^a^	1.59 ^a^	3.29 ^c^	2.09 ^b^	2.08 ^b^	0.082	<0.001

^a,b,c^ Values with different lowercase letters represent significant differences (*p* ≤ 0.05) (*n* = 16).

**Table 7:** GA decreased the area of splenic corpuscle in the broilers.

Treatments							
Items	A	B	C	D	E	SEM ±	*p*-Value
Area of splenic corpuscle (μm^2^)	11,474.12 ^a^	10,161.93 ^a^	18,068.61 ^c^	11,470.99 ^b^	12,059.88 ^b^	720.01	0.003

^a,b,c^ Values with different lowercase letters represent significant differences (*p* ≤ 0.05) (*n* = 3).

**Table 8:** Differences in biochemical indexes between different experimental groups.

Treatments							
Item	A	B	C	D	E	SEM ±	*p*-Value
TP	27.25 ^a^	28.28 ^a^	38.63 ^b^	27.75 ^a^	30.50 ^a^	0.768	<0.001
ALB	14.13 ^a^	14.13 ^a^	17.38 ^b^	14.13 ^a^	14.63 ^a^	0.261	<0.001
GLB	13.13 ^a^	14.00 ^a^	22.88 ^b^	15.25 ^a^	16.75 ^a^	0.696	<0.001
A/G	1.09 ^b^	1.01 ^b^	0.64 ^a^	1.00 ^b^	0.90 ^b^	0.032	<0.001
ALT	2.88 ^a^	3.25 ^a^	10.50 ^b^	4.38 ^a^	4.88 ^a^	0.622	<0.001
AST	185.25 ^a^	205.00 ^a^	701.25 ^d^	462.25 ^c^	332.50 ^b^	33.878	<0.001
ALP	2542.75 ^b^	2795.13 ^b^	1676.13 ^a^	1451.25 ^a^	2528.50 ^b^	98.463	<0.001
TBIL	8.25 ^a^	10.50 ^b^	13.50 ^c^	12.37 ^bc^	10.88 ^a^	0.405	<0.001
Urea	1.31 ^a^	1.20 ^a^	1.69 ^a^	2.15 ^c^	1.48 ^ab^	0.067	<0.001
Cr	30.75 ^a^	31.88 ^a^	77.13 ^b^	32.88 ^a^	40.88 ^a^	3.266	<0.001
UA	281.00 ^a^	282.63 ^a^	448.25 ^b^	260.75 ^a^	300.75 ^a^	18.028	0.003
GLU	15.12	14.74	15.06	14.04	14.16	0.174	0.148
TCHO	3.96 ^a^	4.31 ^a^	6.36 ^c^	5.22 ^a^	4.85 ^a^	0.164	<0.001
ApoB	57.91^b^	55.00 ^b^	38.48 ^a^	42.43 ^a^	56.49 ^b^	2.429	0.016
TG	1.12 ^c^	0.97 ^b,c^	0.53 ^a^	0.64 ^a,b^	0.55 ^a^	0.071	0.017
ApoA-I	59.48 ^c^	56.00 ^c^	36.70 ^a^	47.27 ^b^	47.85 ^b^	1.447	<0.001

^a,b,c,d^ Values with different lowercase letters represent significant differences (*p* ≤ 0.05) (*n* = 8).

## Data Availability

The data in the present study are available on reasonable request from the corresponding author.
